# Bladder squamous cell carcinoma in a pregnant woman: case report and review of the literature

**DOI:** 10.1186/s12894-020-00772-6

**Published:** 2021-01-06

**Authors:** Pablo A. Rojas, Cristián González, Gonzalo P. Mendez, Alejandro Majerson, Ignacio F. San Francisco

**Affiliations:** 1 Departamento de Urología, Escuela de Medicina, Pontificia Universidad Católica de Chile, Diagonal Paraguay 362, Santiago, Chile; 2 División de Ginecología y Obstetricia, Escuela de Medicina, Pontificia Universidad Católica de Chile, Santiago, Chile; 3 Departamento de Anatomía-Patológica, Escuela de Medicina, Pontificia Universidad Católica de Chile, Santiago, Chile

**Keywords:** Bladder cancer, Pregnancy, Radical cystectomy, Case report

## Abstract

**Background:**

Bladder tumors in pregnancy are extremely rare. No more than 50 cases have been published to date, including all histologic variants, and only three cases of bladder squamous cell carcinoma have been described.

**Case presentation:**

We present a clinical case of a 31-year-old woman with bladder squamous cell carcinoma in the second trimester of pregnancy. After a C-section at 30 weeks, we performed radical cystectomy with extended bilateral lymphadenectomy, hysterectomy and right oophorectomy. The Studer neobladder technique was performed for urinary tract reconstruction. Definitive pathology showed invasive bladder squamous cell carcinoma, Grade 2, with microscopic infiltration of the perivesical fat, negative margins, and 3/28 lymph nodes with carcinoma (pT3aN2M0). The patient underwent 18 months of surveillance after radical cystectomy, without recurrence by PET-CT.

**Conclusions:**

Bladder cancer in pregnant women is extremely rare but must be considered in those with recurrent gross hematuria and/or recurrent urinary tract infection. To our knowledge, this case involves the longest recurrence-free survival of a pregnant woman with squamous cell bladder cancer published thus far.

## Background

Bladder tumors inpregnancy are an extremely rare diagnosis. Currently, no more than 50 cases have been published, including all histologic variants [1].

## Case presentation

**Fig. 1:**
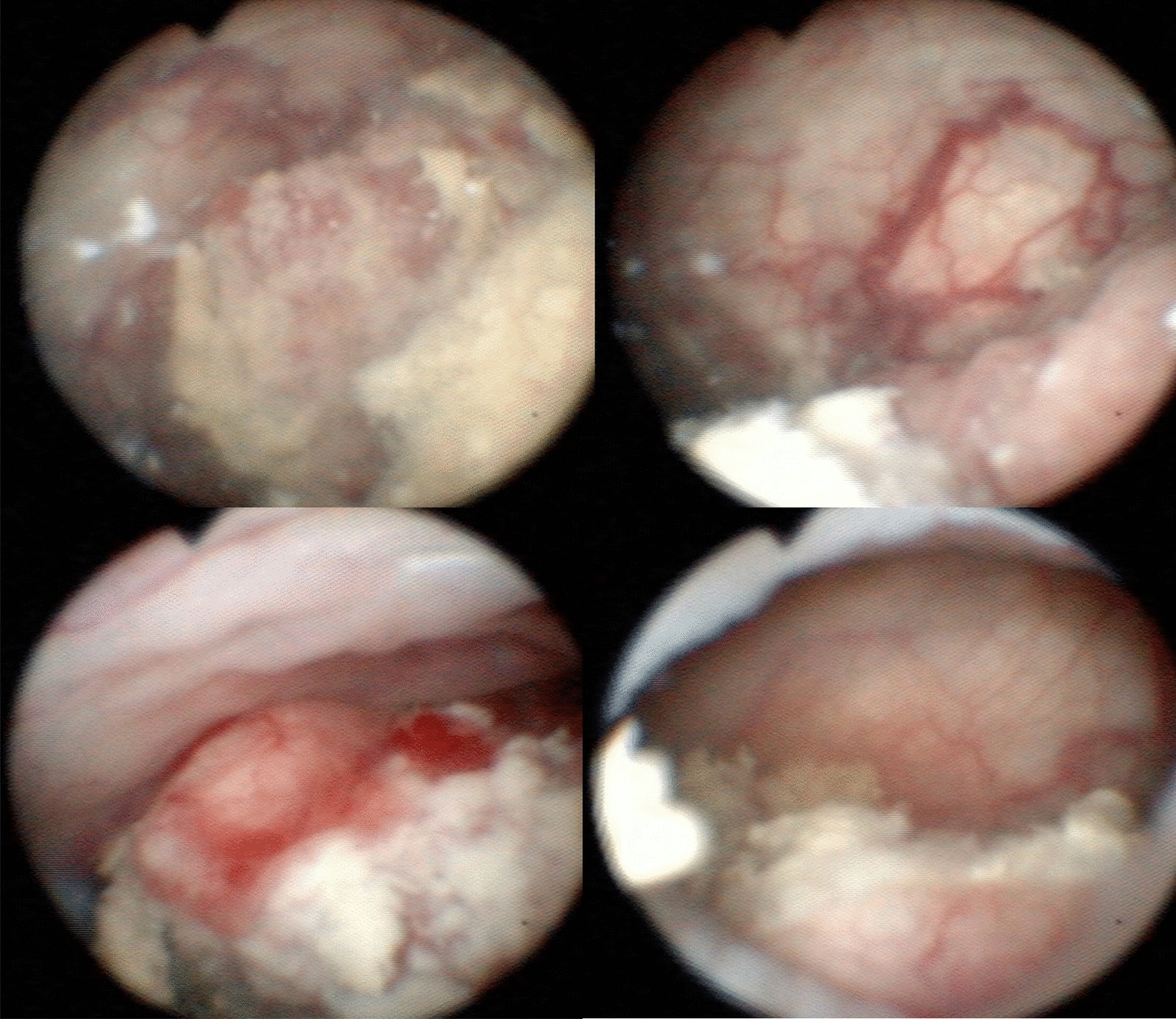
Cystoscopy at 21 weeks of gestation

**Fig. 2:**
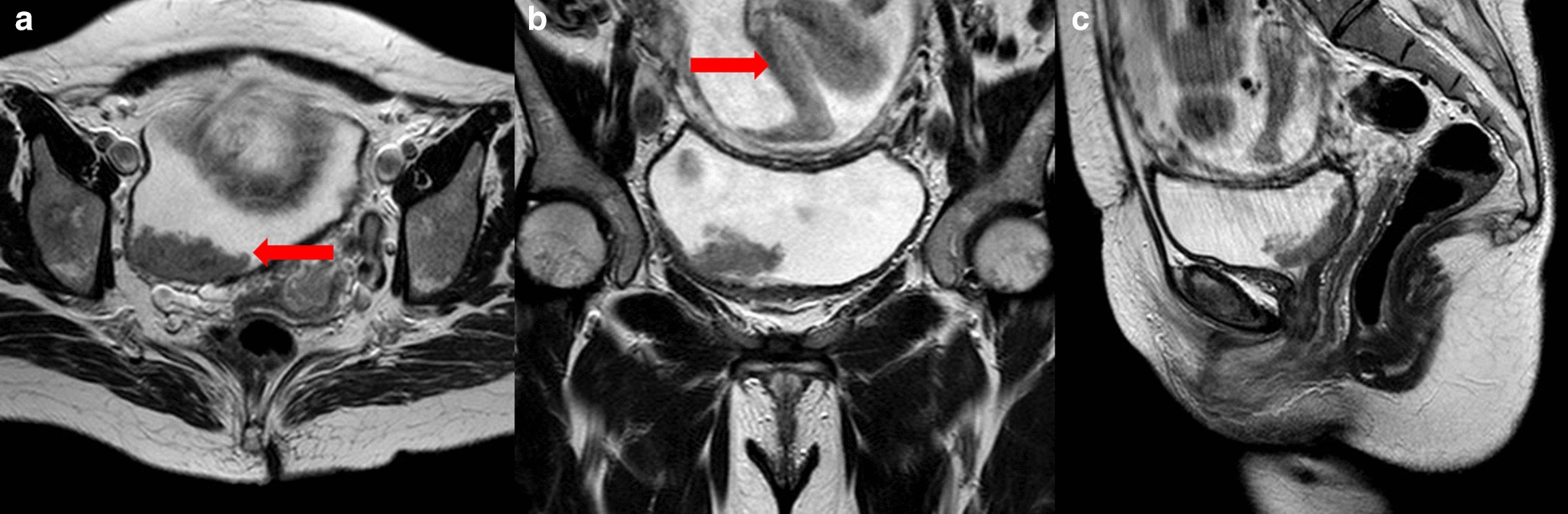
MRI without gadolinium at 23 weeks of gestation. **a** Axial view of the bladder tumor (arrow). **b** Coronal view of the bladder tumor and fetus (arrow). **c** Sagittal view

We present the case of a healthy 31-year-old woman with an obstetric history of 1 pregnancy, 1 live birth, no abortions (G1P1A0), and a urinary tract infection in the first and second trimesters, without complications. At 21 weeks of gestation, the patient had gross hematuria. We performed cystoscopy and observed a lesion of 3–4 cm in the right wall not identifying the right ureteral orifice (Fig. 1). Pelvic magnetic resonance imaging (MRI) without contrast was performed and showed a bladder tumor along the right wall that was 6.3 × 3.5 × 6 cm lateral to the right ureter and lymph nodes, 13 × 9 mm in the right external iliac vessels and 10 × 7 mm in the right internal iliac vessels (Fig. 2). The patient had no predisposing factors for bladder cancer.

From an obstetric perspective, the pregnancy was normal without complications.

At 26 weeks of gestation, we performed transurethral resection (TUR) of the bladder lesion. The biopsy showed well-differentiated, invasive bladder squamous cell carcinoma (pT2). Abdominal MRI and chest computed tomography (CT) did not reveal metastatic disease. A staging study was performed after TUR to evaluate possible tumor dissemination and the need for systemic therapy.

The case was presented to a uro-oncology committee meeting and a maternal fetal medicine unit: the patient was offered the alternative of immediately terminating the pregnancy or waiting until 30 weeks for delivery and subsequently performing the surgery. The patient opted for the latter.

The patient underwent C-section at 30 weeks. The newborn was healthy, with no complications.

As recommended by the multidisciplinary committee, the patient did not receive neoadjuvant chemotherapy based on the histology of pure squamous cell carcinoma. One month after delivery, we performed open radical cystectomy with extended bilateral lymphadenectomy through the common iliac vessels and presacrum, a hysterectomy and a right oophorectomy. Blood loss was 1100 cc. There were no other complications from the surgery. The Studer neobladder technique with 60 cm of terminal ileum was performed for urinary tract reconstruction. Cystostomy was carried out, and a urethral catheter was placed for anastomosis.

The patient recovered satisfactorily and was discharged 5 days postoperatively. The ureteral stents and cystostomy were removed two weeks after surgery.

**Fig. 3:**
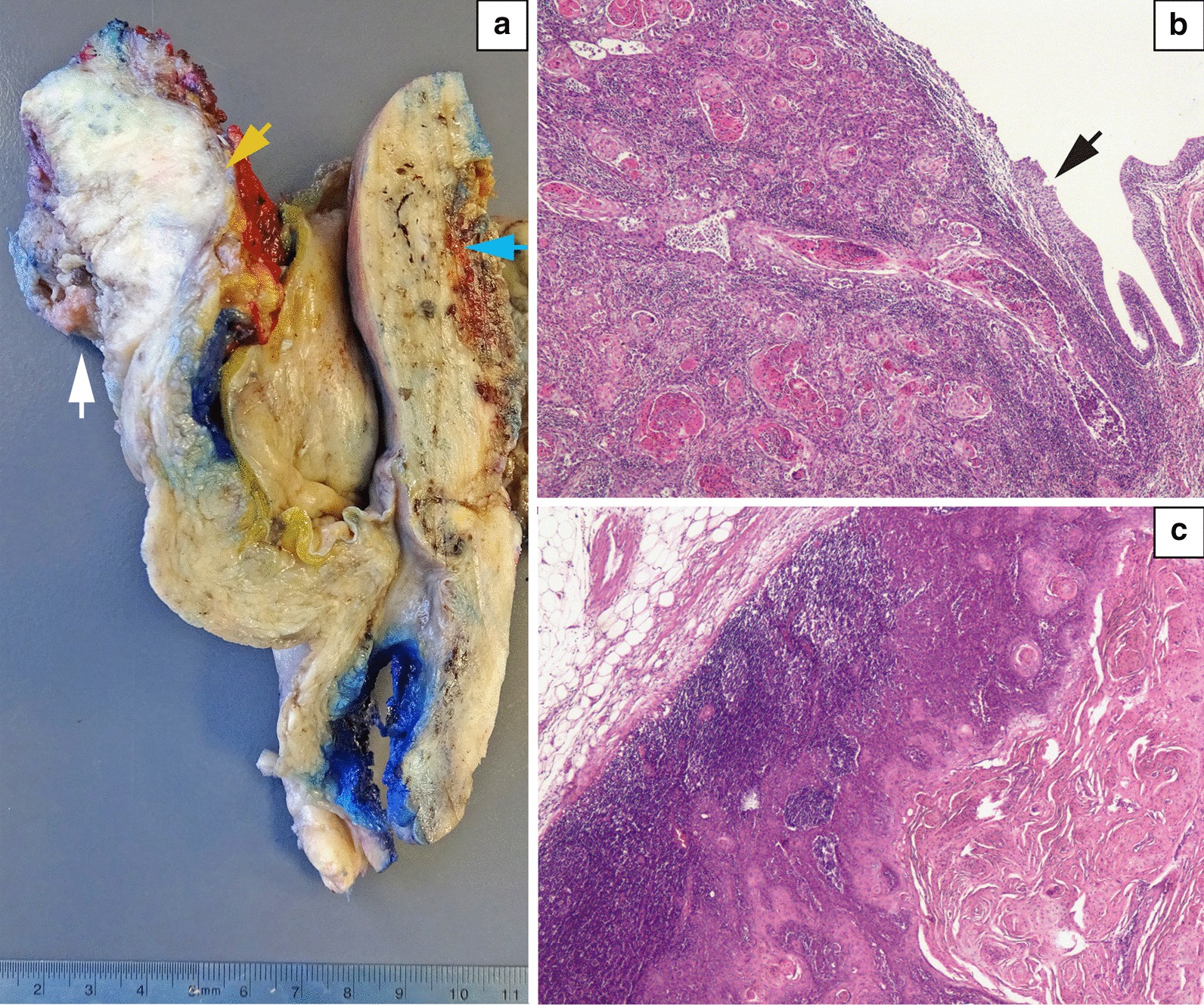
**a** Longitudinal section of the formalin-fixed bladder and uterus. On the left, the bladder mucosa shows a bulky and exophytic, withe-gray tumor (white arrow) that invades through the complete bladder wall, reaching the perivesical adipose tissue (yellow arrow). On the right, the uterine wall and endometrium are thickened (blue arrow), with no evidence of tumor invasion. In between both walls, the vesico-uterine pouch can be recognized. **b** Microphotograph of the bladder mucosa showing a well-differentiated squamous cell carcinoma with multiple keratinizing epithelial groups (pink nests) invading the subepithelial connective tissue. The mucosa has epithelial denudation, and the residual urothelial lining is hyperplastic (arrow). (Hematoxylin and eosin stain, original magnification × 40). **c** Image of one of the iliac lymph nodes with metastatic squamous cell carcinoma (hematoxylin and eosin stain, original magnification × 40)

Definitive pathology revealed invasive bladder squamous cell carcinoma, Grade 2, with microscopic infiltration of the perivesical fat, negative margins, and 3/28 lymph nodes with carcinoma (pT3aN2M0, Fig. 3). Again, evaluation by a multidisciplinary committee decided not to apply adjuvant chemotherapy/immunotherapy or radiation based on the histology and the lack of residual macroscopic disease.

Twenty days after the surgery, the patient presented with abdominal pain and fever. CT with contrast revealed uroperitoneum with neobladder filtration. The patient underwent exploratory laparotomy, which revealed 1200 cc of uroperitoneum, leakage through the cystostomy site, suture line of the ileum and ureterovesical anastomosis. The filtration sites were closed with PDS sutures, and a urethral catheter and drainage were again installed. Five days after the surgery, the patient developed abdominal pain, and an increase in drainage output was observed. Cystography showed new filtration in the anterior wall of the neobladder. The patient again underwent exploratory laparotomy, revealing leakage in two points of the suture line. The filtration sites were closed with PDS sutures. No drainage output or abdominal pain occurred but spontaneous diuresis was present. The patient was discharged at 3 weeks after the first reintervention.

**Fig. 4:**
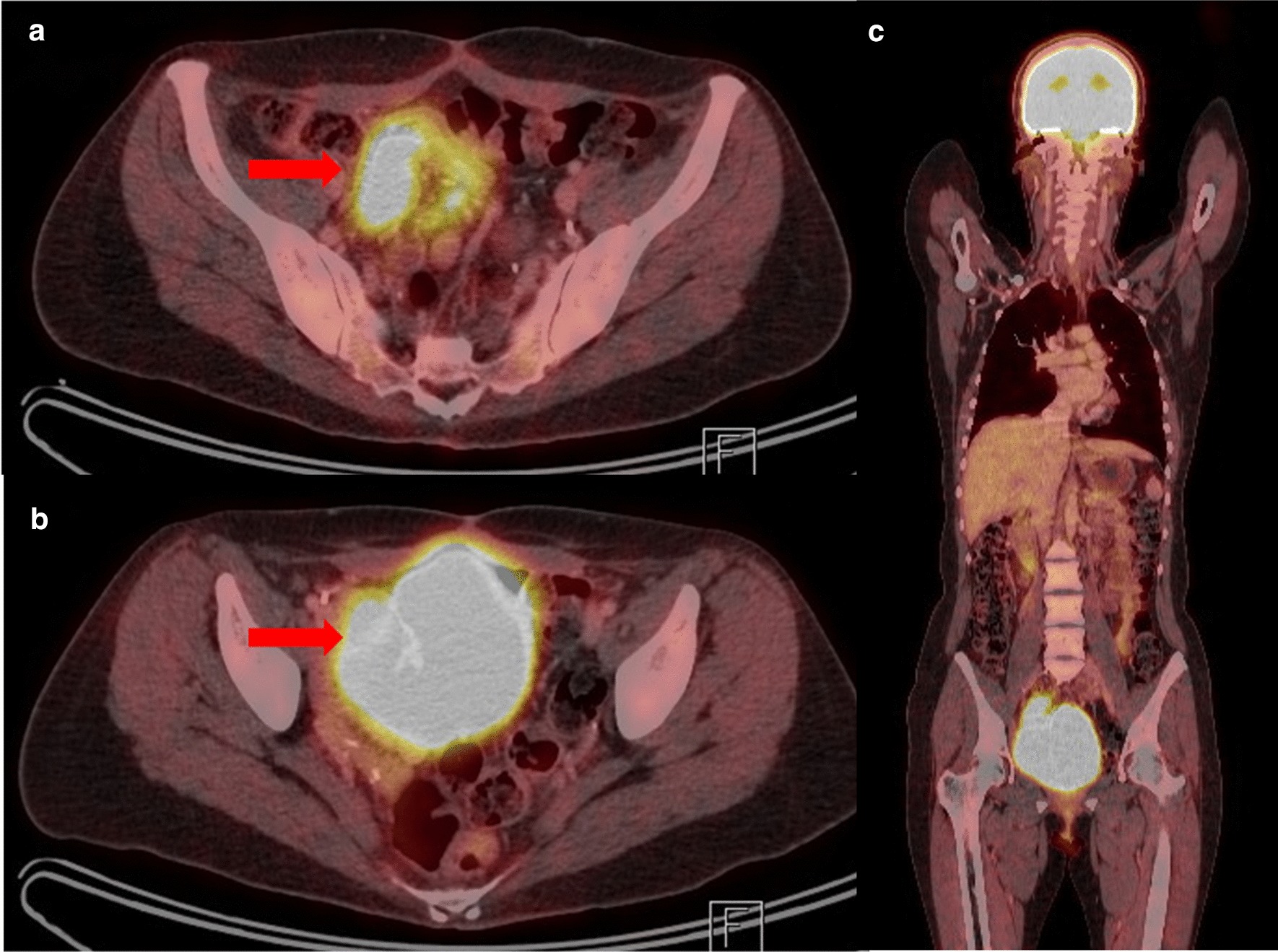
PET-CT with F18-FDG. There was no evidence of locoregional recurrence or distant metastasis after 18 months of surveillance. **a** Axial view of chimney (arrow). **b** axial view of neobladder (arrow). **c** Coronal view

At present, the patient has undergone 18 months of surveillance after radical cystectomy, without recurrence by follow-up PET-CT (Fig. 4), with complete continence, day and night, and no metabolic complications. Her baby is currently 19 months old and perfectly healthy.

## Discussion and conclusions

Urological tumors in pregnant women are exceedingly rare, with an estimated incidence of 0.0013%. The most frequent tumors are kidney tumors, followed by bladder tumors and finally pheochromocytomas [2].

Bladder tumors during pregnancy have been described since 1927, with transitional cell carcinoma being the most common (70%) [3–7]. To our knowledge, only three cases of squamous cell bladder tumors have been reported in pregnant women (Table 1). Risk factors for squamous cell bladder tumors are a history of catheter use (clean intermittent self-catheterization or an indwelling catheter), smoking and recurrent urinary tract infections [8].

**Table 1:** Bladder tumor in pregnant women. Prior studies

Author	Publication’s year	Number of patients	Histology
Church [1]	2013	1 patient, 27 years old	Squamous cell carcinoma
Tyagi [3]	2019	1 patient, 30 years old	Transitional cell carcinoma
Mitra [4]	2003	1 patient, 37 years old	Transitional cell carcinoma
Spahn [6]	2005	3 patients	2 transitional cell carcinoma, 1 squamous cell carcinoma
Alleemudder [7]	2016	1 patient, 39 years old	Squamous cell carcinoma
Shrotri [11]	2008	1 patient, 36 years old	Transitional cell carcinoma

Among reported cases of squamous cell tumors, the women were in the third decade of life with pregnancies in the second trimester, similar to our patient. However, due to the aggressiveness of the disease, patients died within the first year of follow-up after cystectomy [7]. Therefore, to our knowledge, the patient we present in this report has the longest recurrence-free survival described in the literature.

Gross hematuria is frequent in pregnant women (up to 80% can present it), and the most common cause is cystitis [3, 5]; considering the severity of the cases described above, cystoscopy is recommended for pregnant women with recurrent hematuria [7]. MRI can be used as a support tool for diagnosis and staging, and gadolinium can be used from the 23^rd^ week of gestation [2]. An abdomen and pelvis CT scan is not recommended in pregnant women because the fetus will be exposed to radiation above 50 mGy, though a chest CT scan is an alternative to staging because the radiation exposure of the fetus is minimal [9].

Once the diagnosis of the bladder tumor is established, it is essential to perform TUR, which is considered a low-risk procedure at any stage of pregnancy and only carries the usual risks associated with anesthesia [3]. Bipolar TUR may be safer [7]. Using histology, it is possible to categorize patients into risk groups to define management. Low-risk patients should be monitored with cystoscopy. In high-risk patients, a restaging TUR should be performed, and management should be decided according to the histology [2]. The safety of BCG instillations is currently unclear; however, there are reports of their use during the second trimester in a patient with carcinoma in situ [2]. Mitomycin is contraindicated as intravesical therapy during pregnancy due to its teratogenic effects [4].

When establishing that the tumor is infiltrating (pT2), the case must be staged. In addition, the gestation trimester must be considered. In the first and second trimesters, the pregnancy should be terminated, followed by cystectomy [4]; in the third trimester, the recommendation is to wait until 28–30 weeks of gestation and then perform cystectomy (after 3–4 weeks). These recommendations are based on the predicted percentage survival of the fetus, which is only 3% at 22 weeks of gestation but increases to 84% at 28 weeks [4].

Performing both surgeries at once is not recommended, as it would increase the risk of bleeding, considering pelvic congestion and the difficulty of performing a hysterectomy of a pregnant uterus [2]. Regarding urinary diversion, continent and noncontinent diversions have been described, without specific recommendations for the most appropriate surgical technique.

Currently, there is no evidence regarding adjuvant therapy with chemotherapy or systemic immunotherapy in pure squamous cell bladder tumors. Postoperative radiation therapy is an alternative to local control or in cases of positive margins [10].

In conclusion, bladder cancer in pregnant women is extremely rare and must be considered in women with recurrent gross hematuria and/or recurrent urinary tract infection. The management of these cases should be performed by a multidisciplinary team. The best time to perform radical cystectomy seems to be at least 4 weeks after C-section and not at the same time. To our knowledge, this case constitutes the longest recurrence-free survival of a pregnant woman with squamous cell bladder cancer published in the literature.

## Data Availability

Not applicable.

## Abbreviations

MRI: Magnetic resonance imaging
TUTR: Transurethral resection
CT: Computed tomography
PNLMP: Papillary neoplasms of low malignant potential
